# Optimal timing of anticoagulation after acute ischaemic stroke with atrial fibrillation (OPTIMAS): statistical analysis plan for a randomised controlled trial

**DOI:** 10.1186/s13063-025-08761-6

**Published:** 2025-02-19

**Authors:** Norin Ahmed, Hakim-Moulay Dehbi, Nick Freemantle, Jonathan Best, Philip S. Nash, James K. Ruffle, David Doig, David J. Werring

**Affiliations:** 1 Comprehensive Clinical Trials Unit, Institute of Clinical Trials and Methodology, University College London, London, UK; 2 Stroke Research Centre, Department of Brain Repair and Rehabilitation, UCL Queen Square Institute of Neurology, London, UK; 3 UCL Queen Square Institute of Neurology, University College London, London, UK; 4 Department of Neuroradiology, National Hospital for Neurology and Neurosurgery, University College London Hospitals NHS Trust, London, UK

**Keywords:** Statistical analysis plan, Atrial fibrillation, Acute ischaemic stroke, Anticoagulation, DOAC (direct oral anticoagulants), Timing

## Abstract

**Background:**

Atrial fibrillation causes one-fifth of ischaemic strokes, with a high risk of early recurrence. Although long-term anticoagulation is highly effective for stroke prevention in atrial fibrillation, initiation after stroke can be delayed by concerns over intracranial haemorrhage risk. Direct oral anticoagulants offer a significantly lower risk of intracranial haemorrhage than other anticoagulants, potentially allowing earlier anticoagulation and prevention of ischaemic stroke recurrence, but the safety and efficacy of this approach has not been established. This article describes the statistical analysis plan for the OPTIMAS trial as an update to the published protocol. It was written prior to the end of patient follow-up, before database lock and thus while the outcome of the trial is still unknown.

**Aim:**

The optimal timing of anticoagulation after acute ischaemic stroke with atrial fibrillation (OPTIMAS) trial will investigate whether early treatment with a direct oral anticoagulant within 4 days of stroke onset is as effective as, or better than, delayed initiation at 7 to 14 days from onset.

**Methods and design:**

OPTIMAS is a multicentre randomised controlled trial with blinded outcome adjudication. Participants with acute ischaemic stroke and atrial fibrillation eligible for anticoagulation with a direct oral anticoagulant are randomised 1:1 to early or delayed initiation. Here, we describe in detail the statistical aspects of OPTIMAS, including outcome measures, sample size calculation, general analysis principles, descriptive statistics, statistical models, and planned subgroup analyses.

**Study outcomes:**

The primary outcome is a composite of recurrent stroke (ischaemic stroke or symptomatic intracranial haemorrhage) and systemic arterial embolism within 90 days. Secondary outcomes include each individual component of the composite outcome, major bleeding, functional status assessed by the modified Rankin Scale, ongoing anticoagulation, quality of life, health and social care resource use, and length of hospital stay.

**Discussion:**

OPTIMAS aims to provide high-quality evidence on the safety and efficacy of early direct oral anticoagulant initiation after atrial fibrillation-associated acute ischaemic stroke.

**Trial registrations:**

ISRCTN: 17,896,007; ClinicalTrials.gov: NCT03759938. Registered on November 30 2018.

**Supplementary Information:**

The online version contains supplementary material available at 10.1186/s13063-025-08761-6.

## Background

About 20–30% of all ischaemic strokes are associated with atrial fibrillation (AF) [1]. Although the long-term net benefit of oral anticoagulation for stroke prevention in AF is well-established [2, 3], when to start anticoagulation in the acute phase after ischaemic stroke—balancing the risks of early recurrent ischaemic stroke and haemorrhagic transformation of the acute infarct—is a frequent and important dilemma in stroke medicine [2]. Indeed, the first large-scale phase 3 DOAC AF trials excluded patients with recent ischaemic stroke due to concerns about a potential increased risk of intracranial bleeding [4–6].

Although numerous observational studies [7] and two randomised controlled trials [8, 9] have investigated this question, substantial clinical uncertainty remains due to biases and confounding inherent in the observational data and limited statistical power in the randomised trials. Thus, uncertainty remains about the superiority of early anticoagulation and the effects of DOAC timing in key subgroups such as infarct size or stroke severity. In particular, the limited number of participants with moderate-to-severe strokes limits generalisability to the full population of patients with acute ischaemic stroke and atrial fibrillation. The ELAN [9] trial also excluded the key high-risk group of patients taking oral anticoagulation at the time of their stroke, and those with parenchymal haemorrhage types 1 and 2 (but not those with haemorrhagic infarction types 1 and 2).

## Statistical framework

OPTIMAS utilises a gatekeeper approach, investigating whether, in patients with acute ischaemic stroke and AF, early initiation of DOAC treatment (within 4 days (96 h) of onset) is non-inferior to, or better than later initiation of DOAC (Direct (non-vitamin K antagonist [VKA]) oral anticoagulants treatment, no sooner than day 7 (> 144 h) and no later than day 14 (< 336 h) after onset, in preventing recurrent ischaemic stroke, systemic embolism and symptomatic intracranial haemorrhage (sICH). Table 1 below provides our estimand framework with respect to potential intercurrent events (an event of interest occurring after treatment initiation which can affect the interpretation of the endpoint) [10].

**Table 1:** Estimand

Characteristic of estimand (primary outcome)	Definition and method of analysis
Population	Participants aged 18 years or over, clinical diagnosis of acute ischaemic stroke, AF (including paroxysmal, persistent or permanent AF, or atrial flutter). Eligibility to commence DOAC in accordance with approved prescribing recommendations and SmPC confirmed by treating physician and uncertainty on the part of the treating physician regarding early versus standard initiation of DOAC
Treatment conditions	Early (within ≤ 4 days [96 h]) versus standard (between day 7 and day 14 after stroke onset) initiation of anticoagulation after stroke in patients with AF, using any licensed dose of a DOAC
Primary outcome	Composite endpoint of all causes of stroke (i.e. recurrent ischaemic stroke, symptomatic intracranial haemorrhage (including haemorrhagic transformation of the infarct, and strokes that cannot be classified as ischaemic or haemorrhagic due to insufficient data), unclassified stroke syndromes) and systemic arterial embolism at 90 days from randomisation
Summary measure	Difference between study arms in proportion of patients with primary outcome
Intercurrent events handling	
i. DOAC not initiated (clinical or patient decision not to start DOAC)	Treatment policy (intention-to-treat)
ii. DOAC initiated outside of allocated time	Treatment policy (intention-to-treat)
iii. Treatment allocation cross-over	Treatment policy (intention-to-treat)
iv. Death (patient died prior to initiation of DOAC)	While alive (data collected up to point of death)

## Design and methods

### Trial design

OPTIMAS is a large, prospective, partially blinded randomised controlled trial of early (within ≤ 4 days [96 h]) or standard (between day 7 and day 14 after stroke onset) initiation of anticoagulation after stroke in patients with AF, using any licensed dose of a DOAC.

### Randomisation

An independent, concealed, online randomisation service (www.sealedenvelope.com) will be used to minimise allocation bias within the trial. Stratification by stroke severity (NIHSS score at randomisation) comprising five strata will be carried out using random permuted blocks with randomly varying block lengths to ensure that balance across randomisation groups will be achieved. The strata cut-offs for NIHSS will be as follows: 0–4, 5–10, 11–15, 16–21, > 21 [11].

### Sample size

Our originally planned sample size of 3478 patients assumed a reduction in the primary outcome event rate from 11.5% in the control group to 8% in the intervention group. We judged this to be a clinically meaningful benefit likely to influence guidelines and practice. The assumed composite event rate and hypothesised effect size were derived from the Virtual International Stroke Trials Archive of trials in patients with ischaemic stroke and AF [7]. The sample size calculation used 90% power for superiority, significance level 5%, and was inflated by 10% for loss to follow-up.

We re-evaluated study power in November 2021 due to a lower-than-expected interim adjudicated primary outcome rate of 4.3% averaged across both treatment arms. Data published in 2022 from a comparable trial, TIMING [8], showed a reduced rate of ischaemic stroke from 4.57 to 3.11%. This corresponds to an odds ratio of 0.67 (95% CI: 0.33 to 1.35).

The ELAN [9] trial, published in 2023, also found a reduced rate of ischaemic stroke at 90 days in the early compared to the later treatment arm (1.9% compared to 3.1%, odds ratio, 0.60; 95% CI, 0.33 to 1.06) [9]. There was no difference in the rate of intracranial haemorrhage at 90 days (0.2% in both arms). In ELAN, the rate of a similar composite outcome to that used in OPTMAS was 24/968 (2.48%) in the early arm compared to 42/965 (4.35%) in the later arm [9].

With the observed lower event rate of 4.3%, our planned sample size has 80% power to show non-inferiority based on an absolute non-inferiority margin of 2% (i.e. 2 percentage points) assuming an equal rate of 4.3% in both arms and one-sided alpha of 5%, and 80% power for superiority assuming an odds ratio of 0.62 with an assumed event rate in the control arm of 5.3%.

### Analysis framework

OPTIMAS will use a non-inferiority gatekeeper approach to test for non-inferiority of early anticoagulation followed by a test for superiority if non-inferiority is established.

No formal interim analysis is planned within the RCT. Participant safety will be assessed by an Independent Data Monitoring Committee which will have untrammelled access to the study data in order to advise the sponsor on potential harm to subjects.

### Timing of final analysis

The final analysis will start when all data for the primary endpoint are entered into the database and all corresponding queries are resolved. At this point, the database will be locked, which means that no data can be entered and/or modified.

### Confidence intervals and *p*-values

All applicable statistical tests will be 2-sided and will be performed using a 5% significance level, unless otherwise specified. All confidence intervals presented will be 95% and two-sided.

### Analysis population

The primary analysis will be conducted following the modified intention-to-treat (mITT) principle in accordance with the randomised intervention. The analysis will include all participants randomised into the trial, except those subsequently found not to have AF and/or not to have a confirmed acute ischaemic stroke.

Participants who are lost to follow-up with respect to binary endpoints within the 90-day timeframe (e.g. the primary endpoint and its components) will be included in the primary mITT analysis, with the assumption that for these participants the event did not take place. For patients who withdrew their consent for further follow-up, the analysis will include them, with the assumption that the event did not take place. For patients who died during the 90-day follow-up without experiencing the primary endpoint, these will be included in the mITT analysis. The reasoning described in this paragraph also applies to binary endpoints within the 30-day timeframe.

### Trial population

#### Patient eligibility criteria

Inclusion criteria:

1. Aged 18 years or over
2. Clinical diagnosis of acute ischaemic stroke
3. AF (including paroxysmal, persistent or permanent AF, or atrial flutter), confirmed by any of:
  - 12-lead ECG recording
  - Inpatient ECG telemetry
  - Documentation of a diagnosis of AF detected before or after the acute ischaemic stroke in medical records (e.g. primary care records, letter from secondary care)
4. Eligibility to commence DOAC in accordance with approved prescribing recommendations and SmPC confirmed by treating physician.
5. Uncertainty on the part of the treating physician regarding early versus standard initiation of DOAC.

Exclusion criteria:

1. Contraindication to anticoagulation:
  - Known coagulopathy or current or recent anticoagulation with vitamin K antagonist (VKA) leading to INR ≥ 1.7 at randomisation.
  - Thrombocytopenia (platelets < 75 × 109/L)
  - Other coagulopathy or bleeding tendency (based on clinical history or laboratory parameters) judged to contraindicate anticoagulation by treating clinician
2. Contraindication to early anticoagulation
  - Known presence of haemorrhagic transformation with parenchymal haematoma occupying > 30% of the infarct volume and exerting significant mass effect (i.e. PH2) (NB: HI1, HI2 and PH1 are not considered contraindications), based on Heidelberg criteria [12]
  - Presence of clinically significant intracranial haemorrhage unrelated to qualifying infarct
  - Any other contraindication to early anticoagulation as judged by the treating clinician
3. Contraindication to use of DOAC:
  - Known allergy or intolerance to both factor Xa inhibitor and direct thrombin inhibitor
  - Definite indication for VKA treatment e.g. mechanical heart valve, valvular AF (determined by the local investigator, but usually defined as moderate to severe mitral valve disease, a prosthetic valve, or both), antiphospholipid syndrome
  - Severe renal impairment with creatinine clearance (Cockcroft & Gault formula) < 15 mL/min (i.e. 14 mL/min or less)
  - Liver function tests ALT > 2 × ULN
  - Cirrhotic patients with Child–Pugh score equating to grade B or C
  - Patient is taking medication with significant interaction with DOAC, including:
    Azole antifungals (e.g. ketoconazole, itraconazole)
    HIV protease inhibitors (e.g. ritonavir)
    Strong CYP3A4 inducers (e.g. rifampicin, phenytoin, carbamazepine, phenobarbital or St. John’s Wort)
    Dronedarone
4. Pregnant or breastfeeding women
5. Presence on acute brain imaging of non-stroke pathology judged likely to explain clinical presentation (e.g. mass lesion, encephalitis)
6. Inability for patient to be followed up within 90 days of trial entry
7. Patient or designated representative(s) (according to relevant legislation for each nation) refusal to consent to study procedures, including the site informing GP and healthcare professional responsible for anticoagulation care of participants
8. Any other reason that the PI considers would make the patient unsuitable to enter OPTIMAS.

#### Recruitment, follow-up and withdrawal

Recruitment will be presented by year and centre. Participants will be followed up for 90 days from randomisation. The throughput of patients from those screened, those randomised and those assessed at each visit and included in the analysis will be summarised in a CONSORT flowchart. The number of patients who withdraw and are unwilling to provide follow-up will be reported by the treatment arm.

#### Baseline patient characteristics

Baseline characteristics include demographics such as sex, age and race, as well as details of presenting stroke, clinical presentation and medical history. Other baseline assessments include estimated pre-stroke mRS (a measure of function and dependence for mobility and self-care), IQCODE (a measure of pre-stroke cognitive function) and EQ-5D-5L (a measure of health-related quality of life) scores. Baseline characteristics will be summarised by the treatment arm using appropriate descriptive statistics; means and standard deviations for approximately normally distributed variables, medians and interquartile ranges for non-normally distributed variables and counts (percentages) for categorical variables. The variables to be reported in the baseline tables are listed in the dummy tables (Additional file 1, Table A1).

### Outcome definitions

#### Primary outcome

The primary outcome is a binary classification of those participants who experience the composite endpoint of all causes of stroke (i.e. recurrent ischaemic stroke, symptomatic intracranial haemorrhage (including haemorrhagic transformation of the infarct), and strokes that cannot be classified as ischaemic or haemorrhagic due to insufficient data), unclassified stroke syndromes and systemic arterial embolism, and those who do not experience any of the composite outcome events, within 90 days from randomisation.

#### Secondary outcomes

All secondary outcomes are within 90 days of randomisation (unless stated otherwise).

#### Efficacy outcomes

- All-cause mortality
- Vascular death
- Recurrent ischaemic stroke
- Systemic arterial embolism
- Venous thromboembolism (deep vein thrombosis [DVT], pulmonary embolism [PE], cerebral venous thrombosis [CVT])
- Functional status (assessed by the mRS scale)
- Cognitive ability (assessed by the MoCA questionnaire)
- Quality of life at 90 days (assessed by EQ-5D 5 level [EQ-5D-5L])
- Patient-reported outcomes (assessed by the PROMIS-10)
- Ongoing anticoagulation
- Time to event for overall survival
- Time to first incidence of composite primary outcome plus overall survival
- Time to first incidence of composite primary outcome (recurrent ischaemic stroke, systemic arterial embolism, sICH or unclassifiable stroke syndromes)
- Time to first incidence composite of the ischaemic components of the primary outcome (ischaemic stroke and systemic embolism)
- Time to first incidence of symptomatic ICH
- Time to first incidence of ischaemic stroke
- Time to first incidence of systemic arterial embolism
- Length of hospital stay for stroke-related care
- Health and Social Care Resources (assessed by a study-specific questionnaire: HSCR).

#### Safety outcomes

- sICH at 90 days, classified according to site: intracerebral haemorrhage (within the brain parenchyma); subdural haemorrhage; extradural haemorrhage; subarachnoid haemorrhage; and haemorrhagic transformation of a brain infarct
- Incidence of major extracranial bleeding
- Incidence of all major bleeding (intracranial and extracranial)
- Incidence of clinically relevant non-major bleeding

#### Exploratory outcomes

- Individual cognitive domain sub-scores (measured using the MoCA questionnaire).

### Rationale and details for outcome measures

In AF-associated acute ischaemic stroke, the risk of early recurrence (within 7–14 days) is high, between 0.4% and 1.3% per day [13, 14]. AF-associated ischaemic strokes are more often disabling or fatal than other types of stroke, with longer hospital stays and higher costs [15], so preventing early recurrence is a key clinical challenge. Anticoagulation is extremely effective for long-term AF stroke prevention, [16] but safety and benefit in acute stroke has not been established. Early anticoagulation (i.e. in the first few days) might increase the risk of symptomatic intracranial haemorrhage (ICH), including haemorrhagic transformation of the infarct (estimated at ~ 1% per day [17], leading to clinical uncertainty about when to start anticoagulation. Observational studies reported an 8–10% risk of recurrent ischaemic stroke and a 2–4% risk of symptomatic intracranial haemorrhage within 90 days of AF-associated ischaemic stroke [18, 19].

DOACs—apixaban, dabigatran, edoxaban or rivaroxaban—have a 50% lower risk of intracranial haemorrhage compared to VKA [20]. DOACs might allow safe and earlier oral anticoagulation after acute ischaemic stroke in patients with AF, providing net benefit by reducing ischaemic stroke recurrence without increased ICH risk.

We chose a composite primary outcome of all strokes (due to both ischaemia or intracranial bleeding) and systemic embolism as these events are those most likely to be modified by the timing of anticoagulation and to be most clinically relevant for patients and treating clinicians.

### Analysis methods

The results of the analyses will be reported following the principle of the ICH E3 guidelines on the Structure and Content of Clinical Study Reports [21]. Dummy tables are presented in the Appendix. All analysis will be performed on statistical software STATA 18 MP or the latest version available at the time of analysis.

### Adjustment factors

The primary outcome model will be adjusted by the stratifying variable for stroke severity at randomisation (NIHSS).

### Primary outcome analysis

The primary outcome is the composite endpoint of all causes of stroke, i.e. recurrent ischaemic stroke, symptomatic intracranial haemorrhage (including haemorrhagic transformation of the qualifying infarct, unclassified strokes (cannot be classified as ischaemic or haemorrhagic due to insufficient data) and systemic arterial embolism at 90 days from randomisation. We will test for any difference in the proportion of patients, according to the treatment arm, who do or do not experience the primary outcome.

Since we are using a gatekeeper approach, the first stage of the analysis of the primary outcome will be to establish non-inferiority. Our non-inferiority margin of 2 percentage points is based on a conventional non-inferiority power calculation with our original target sample size of 3478. The non-inferiority margin set in the TIMING [8] trial was an absolute difference of 3 percentage points. In our judgement, this is a large difference given contemporary data on event rates (e.g. a total event rate for our primary outcome of 66/1933 3.41% in ELAN [9]). We regard our original non-inferiority margin of 2 points to be reasonable because an absolute risk increase in our primary outcome of 2% from the baseline event rate of 3–4% is likely to be considered clinically important and discouraging for the use of early DOAC. If the non-inferiority condition is met, such that early initiation of DOAC is found to be non-inferior to standard initiation in preventing the primary outcome event, we will then test for superiority of the intervention compared to control.

The primary outcome has a binary classification in which a participant has either experienced at least 1 or more of the individual primary endpoints or has not experienced any of them. When a participant experiences their first event this will be included in the primary analysis and will then be censored hereafter for any subsequent events reported. Mixed effects logistic regression will be used to determine whether there is any difference in the risk of a primary outcome event using the treatment arm as a binary covariate. The results will be adjusted for the stratifying variable, the NIHSS score at randomisation, which will be included as an additional covariate in the model. Sites will be included as random effects.

Should there be any issues with model convergence, standard logistic regression will be used. In this case, it will not be possible to account for variability between sites, therefore we plan to produce a fixed effects model with and without site as an adjustment factor and compare the models. The model coefficient due to treatment will give an estimate of the difference in log odds between the treatment arms accounting for stroke severity.

### Secondary outcome analysis (excluding Health Economic Outcomes)

Secondary and exploratory outcomes will be handled similarly with adjustment for the stratifying variable (stroke severity) and with sites included as random effects. All secondary outcomes are within 90 days of randomisation.

#### Binary outcomes

Binary secondary outcomes will be analysed using mixed effects logistic regression models.

#### Continuous outcomes

Continuous outcome measures will be analysed using mixed effects linear regression models.

#### Time to event outcomes

Time-to-event outcomes will be described using Kaplan–Meier plots and Cox proportional hazards models will be used to obtain hazard ratios. In case of evidence that the proportional hazards assumption is not met, we will use a stratified log-rank test.

#### Categorical outcomes

Ordered categorical outcomes will be analysed using a mixed effects ordinal logistic regression model.

#### Adverse events

Adverse events will be summarised in terms of the number of (serious) adverse events and the number of participants with any (serious) adverse events in each randomised group and compared using Fisher’s exact test. The difference (in the number of serious adverse events in each randomised group) and *p*-value will be presented.

### Subgroup analysis

Results of the primary outcome will be presented by stratum, i.e. according to the levels of stroke severity at randomisation, the only stratifying variable used in the randomisation process. An interaction between the subgroup and treatment will be added to the primary analysis model to investigate whether the treatment effect differs according to the levels of stroke severity (NIHSS 0–4, 5–10, 11–15, 16–21, > 21). As the trial has not been powered to detect this, this should be considered a supportive analysis.

Further hypothesis-generating subgroup analyses will be undertaken for the following groups, describing the effects within each stratum and the interaction between these and early versus late DOAC:

- Age (< 75 years vs ≥ 75 years)
- Sex (male/female)
- Use of anticoagulation at the time of qualifying acute ischaemic stroke (yes/no)
- Atrial fibrillation previously known before qualifying acute ischaemic stroke (yes/no)
- Diabetes mellitus (yes/no)
- Known chronic kidney disease (yes/no)
- Reperfusion therapy (none/intravenous thrombolysis alone/intravenous thrombolysis and mechanical thrombectomy/mechanical thrombectomy alone)

### Neuroimaging substudy analysis

We will undertake a pre-specified imaging substudy to determine whether any neuroimaging features modify the primary results of the trial. We will undertake these analyses on both CT scans (for all participants) and MRI scans where available.

The primary imaging exposure of interest will be infarct volume. We will investigate whether this modifies the treatment effect (of early or late anticoagulation) by including the volume as an interaction term with the treatment allocation in the mixed effects logistic regression models.

As secondary analyses of the neuroimaging substudy, we will investigate whether additional imaging variables modify the treatment effect by including them as interaction terms in the same way. Other imaging subgroups [22] will include:

Subgroups will include:

- Infarct size (minor, moderate, severe, using the same classification as the ELAN [9] trial
- Infarct location (anterior vs posterior circulation)
- Haemorrhagic transformation (none, petechial (HI1, HI2), parenchymal (PH1, PH2), or remote)
- Presence of severe cerebral small vessel disease (leukoaraiosis) on CT (yes/no)
- Presence of severe cerebral small vessel disease (leukoaraiosis) on MRI (yes/no)
- Number of cerebral microbleeds on MRI, categorised as 0, 1, 2–4, and 5 or more.

A full description of the planned neuroimaging substudies is provided in the Appendix.

### Missing data

Missing data on the primary outcome is expected to be less than 10% based on previous studies in this area. The primary outcome is achieved (or not) at 90 days during which time the participant population is expected to maintain close ties with health services during their stroke rehabilitation period. Missing covariate data are not anticipated since covariates must be recorded to allocate treatment. The primary analysis is likelihood based and therefore robust to the assumption of missing-at-random, and missing observations in primary and secondary outcomes will not be imputed, but should substantial missing data be encountered, the reasons for missingness will be investigated using logistic regression of covariates on an indicator of missingness to help inform the interpretation of the trial results.

A sensitivity analysis for the primary endpoint and its components will be undertaken, where patients lost-to-follow-up and patients who have withdrawn their consent for further follow-up will be excluded.

## Discussion

This article contains the pre-specified statistical analysis plan for the OPTIMAS trial. By publishing the statistical analysis plan we aim to increase the transparency of the data analysis. The OPTIMAS trial will provide high-quality evidence on the safety and efficacy of early direct oral anticoagulant initiation after atrial fibrillation-associated ischaemic stroke.

## Supplementary Information

Additional file 1. Dummy Tables. This file contains dummy tables which show the planned format and contents of the tables for the OPTIMAS final statistical report.

Additional file 2. Neuroimaging Substudy Analysis Plan. This file contains the analysis plan for the Neuroimaging substudy which will use the results of the data collected from the OPTIMAS trial for secondary analyses

Additional file 3. OPTIMAS Trial Team and Oversight Committee Members. This file contains the full list of OPTIMAS trial team and oversight committee members including names, affiliations and role. Statistical Analysis Plan (SAP) Checklist v1.0

## Data Availability

The protocol has previously been published^9^. Following completion of the trial analysis, the results will be published, and additional available data can be obtained by contacting the chief investigator (DJW). The study team retain exclusive use until the publication of major outputs has been completed.

## Abbreviations

AE: Adverse event
AF: Atrial fibrillation
AR: Adverse reaction
ASU: Acute stroke unit
CA: Competent authority
CCTU: Comprehensive Clinical Trials Unit
CI: Chief Investigator
CRF: Case Report Form
CSRI: Client Service Receipt Inventory
CT: Computed tomography
CTA: Clinical Trial Authorisation
CTCAE: Common Terminology Criteria for Adverse Events
CV: Curriculum Vitae
CVT: Cerebral venous thrombosis
DOAC: Direct Oral Anticoagulant
DVT: Deep vein thrombosis
EC: Ethics Committee
EU: European Union
EAC: Event Adjudication Committee
ECASS: European Cooperative Acute Stroke Study
EQ-5D-5L: EuroQol EQ-5D 5 level
HSCIC: Health & Social Care Information Centre
HEAP: Health Economic Analysis Plan
HES: Hospital Episode Statistics
ICH GCP: International Conference on Harmonisation Good Clinical Practice
GDPR: General Data Protection Regulation
GP: General Practitioner
HASU: Hyperacute stroke unit
HDU: High-dependency unit
HSCR: Health and Social Care Resources questionnaire
ICF: Informed consent form
ICH: Intracerebral haemorrhage
IDMC: Independent Data Monitoring Committee
INR: International normalised ratio
IQCODE: Informant Questionnaire on Cognitive Decline in the Elderly
ISF: Investigator Site File
ISTH: International Society on Thrombosis and Haemostasis
ITT: Intention-to-treat
mITT: Modified intention-to-treat
MDT: Multidisciplinary team
mRS: Modified Rankin scale
MHRA: Medicines and Healthcare products Regulatory Agency
MoCA: The Montreal Cognitive Assessment
MRI: Magnetic resonance imaging
NAE: Notifiable adverse event
NHS: National Health Service
NHS IEP: NHS Imaging Exchange Portal
NIHSS: National Institute of Health Stroke Scale
NOAC: Non-vitamin K antagonist oral anticoagulants; DOAC and NOAC are interchangeable terms
Non-CTIMP: Trials that do not involve an Investigational Medicinal Product
OAC: Oral anticoagulant
PE: Pulmonary embolism
PI: Principal Investigator
PIS: Participant Information Sheet
PROMIS—10: Patient-Reported Outcomes Measurement Information System
QA: Quality assurance
QC: Quality control
QMMP: Quality Management and Monitoring Plan
R&D: Research and Development
REC: Research Ethics Committee
SAE: Serious adverse event
SAP: Statistical analysis plan
SAR: Serious adverse reaction
sICH: Symptomatic intracranial haemorrhage
SmPC: Summary of product characteristics
SSA: Site-specific approval
TIA: Transient ischaemic attack
TMF: Trial Master File
TMG: Trial Management Group
TMT: Trial Management Team
ToR: Terms of Reference
TSC: Trial Steering Committee
UCL: University College London
USM: Urgent Safety Measure
VKA: Vitamin K antagonist
