# Supplementary material for: Optimal timing of anticoagulation after acute ischaemic stroke with atrial fibrillation (OPTIMAS): statistical analysis plan for a randomised controlled trial
Source: Trials. 2025 Feb 19;26:58. doi: 10.1186/s13063-025-08761-6 (PMC11837694; doi:10.1186/s13063-025-08761-6)
Supplement: Supplementary file 1 — Additional file 1. Dummy Tables. This file contains dummy tables which show the planned format and contents of the tables for the OPTIMAS final statistical report. [file 13063_2025_8761_MOESM1_ESM.docx]

**Additional File 1 – Dummy Tables**

**Table A1: Baseline Characteristics**

| **Baseline Characteristic** | **Early n=** | **Standard n=** | **Total N=** |
| --- | --- | --- | --- |
| Age (years); mean (sd) |  |  |  |
| Estimated pre-stroke mRS score; mean (sd) |  |  |  |
| Sex |  |  |  |
| Female; n (%) |  |  |  |
| Male; n (%) |  |  |  |
| Ethnicity |  |  |  |
| *Caucasian; n (%) |  |  |  |
| **All Other Ethnic Groups; n (%) |  |  |  |
| Not Stated; n (%) |  |  |  |
| Anticoagulant/Antiplatelet prior to admission |  |  |  |
| No; n (%) |  |  |  |
| Yes; n (%) |  |  |  |
| Was/is the participant on warfarin? |  |  |  |
| No; n (%) |  |  |  |
| Yes; n (%) |  |  |  |
| Was/is the participant taking a DOAC? |  |  |  |
| No; n (%) |  |  |  |
| Yes; n (%) |  |  |  |
| IV Thrombolysis |  |  |  |
| No; n (%) |  |  |  |
| Yes; n (%) |  |  |  |
| Endovascular treatment |  |  |  |
| No; n (%) |  |  |  |
| Yes; n (%) |  |  |  |
| Hypercholesterolemia |  |  |  |
| No; n (%) |  |  |  |
| Yes; n (%) |  |  |  |
| Not Known; n (%) |  |  |  |
| Diabetes type 1 or 2, known prior to stroke or diagnosis |  |  |  |
| No; n (%) |  |  |  |
| Yes; n (%) |  |  |  |
| Not Known; n (%) |  |  |  |
| Hypertension |  |  |  |
| No; n (%) |  |  |  |
| Yes; n (%) |  |  |  |
| Not Known; n (%) |  |  |  |
| Known chronic kidney disease |  |  |  |
| No; n (%) |  |  |  |
| Yes; n (%) |  |  |  |
| Not Known; n (%) |  |  |  |
| Known dementia/cognitive impairment |  |  |  |
| No; n (%) |  |  |  |
| Yes; n (%) |  |  |  |
| Not Known; n (%) |  |  |  |
| Smoker status |  |  |  |
| Current smoker; n (%) |  |  |  |
| Ex smoker; n (%) |  |  |  |
| Never smoked; n (%) |  |  |  |
| Not Known; n (%) |  |  |  |
| Current alcohol intake >14 units per week |  |  |  |
| No; n (%) |  |  |  |
| Yes; n (%) |  |  |  |
| Not Known; n (%) |  |  |  |
| Myocardial infarction |  |  |  |
| No; n (%) |  |  |  |
| Yes; n (%) |  |  |  |
| Not Known; n (%) |  |  |  |
| Coronary Revascularisation |  |  |  |
| No; n (%) |  |  |  |
| Yes; n (%) |  |  |  |
| Not Known; n (%) |  |  |  |
| Congestive heart failure |  |  |  |
| No; n (%) |  |  |  |
| Yes; n (%) |  |  |  |
| Not Known; n (%) |  |  |  |
| History of angina |  |  |  |
| No; n (%) |  |  |  |
| Yes; n (%) |  |  |  |
| Not Known; n (%) |  |  |  |
| Peripheral arterial disease |  |  |  |
| No; n (%) |  |  |  |
| Yes; n (%) |  |  |  |
| Not Known; n (%) |  |  |  |
| Previous ischaemic stroke |  |  |  |
| No; n (%) |  |  |  |
| Yes; n (%) |  |  |  |
| Previous other intracranial bleeding |  |  |  |
| No; n (%) |  |  |  |
| Yes; n (%) |  |  |  |
| Type of Atrial fibrillation |  |  |  |
| Paroxysmal; n (%) |  |  |  |
| Persistent; n (%) |  |  |  |
| Previous hospitalisation for extracranial bleeding? |  |  |  |
| No; n (%) |  |  |  |
| Yes; n (%) |  |  |  |
| Stroke severity (NIHSS) at Admission |  |  |  |
| 0-4; n (%) |  |  |  |
| 5-10; n (%) |  |  |  |
| 11-15; n (%) |  |  |  |
| 16-21; n (%) |  |  |  |
| >21; n (%) |  |  |  |
| Stroke severity (NIHSS) at Randomisation |  |  |  |
| 0-4; n (%) |  |  |  |
| 5-10; n (%) |  |  |  |
| 11-15; n (%) |  |  |  |
| 16-21; n (%) |  |  |  |
| >21; n (%) |  |  |  |

**Caucasian include White British, White Other and White Irish*

***All Other Ethnic Groups include Black, Asian and other ethnic minorities (BAME)*

**Table A2: Primary outcome**

|  | **Early n=** | **Standard n=** | **Adjusted Risk Difference (95% CI)** | **p-value** |
| --- | --- | --- | --- | --- |
| **Composite primary outcome total; n (%)** |  |  |  |  |

**Composite primary outcome includes the number of participants experiencing at least 1 or more of the composite endpoints, including stroke of any cause (recurrent symptomatic ischaemic stroke, symptomatic intracranial haemorrhage, unclassified stroke syndromes) and/or systemic arterial embolism, within the 90 days following randomisation.*

**Table A3: Secondary outcomes**

| **Secondary Outcome** | **Early n=** | **Standard n=** | **Adjusted Risk Difference (95% CI)** | **p-value** |
| --- | --- | --- | --- | --- |
| Recurrent ischaemic stroke; n (%) |  |  |  |  |
| Symptomatic intracranial haemorrhage (sICH); n (%) |  |  |  |  |
| Unclassifiable stroke syndromes; n (%) |  |  |  |  |
| Systemic arterial embolism; n (%) |  |  |  |  |
| All-cause mortality; n (%) |  |  |  |  |
| Vascular death; n (%) |  |  |  |  |
| Venous thromboembolism; n (%) |  |  |  |  |
| Functional status (assessed by the mRS scale); n (%) |  |  |  |  |
| Ongoing anticoagulation at 90 days from randomisation; n (%) |  |  |  |  |
|  |  |  | **Adjusted coefficient (95% CI)** | **p-value** |
| Patient reported outcomes (assessed by the PROMIS-10) |  |  |  |  |
| Physical health); mean (sd) |  |  |  |  |
| Mental health); mean (sd) |  |  |  |  |
|  |  |  |  |  |
| Length of hospital stay for stroke-related care); mean (sd) |  |  |  |  |
| Individual cognitive domain subscores (MoCA); mean (sd) |  |  |  |  |
| Overall MoCA; mean (sd) |  |  |  |  |
|  |  |  | **Adjusted Hazard Ratio (95% CI)** | **p-value** |
| Time to event for overall survival; med (IQR) |  |  |  |  |
| Time to first incidence of composite primary outcome plus overall survival; med (IQR) |  |  |  |  |
| Time to first incidence of composite primary outcome; med (IQR) |  |  |  |  |
| Time to first incidence composite of the ischaemic components of the primary outcome (ischaemic stroke and systemic embolism); med (IQR) |  |  |  |  |
| Time to first incidence of symptomatic ICH; med (IQR) |  |  |  |  |
| Time to first incidence ischaemic stroke; med (IQR) |  |  |  |  |
| Time to first incidence systemic arterial embolism; med (IQR) |  |  |  |  |

*med = median, IQR = interquartile range*

**Table A4: Safety Outcomes**

| **Safety Outcome** | **Early n=** | **Standard n=** | **Adjusted Risk Difference (95% CI)** | **p-value** |
| --- | --- | --- | --- | --- |
| Symptomatic intracranial haemorrhage (sICH) according to site: |  |  |  |  |
| intracerebral haemorrhage (within the brain parenchyma); n (%) |  |  |  |  |
| subdural haemorrhage; n (%) |  |  |  |  |
| extradural haemorrhage; n (%) |  |  |  |  |
| subarachnoid haemorrhage; n (%) |  |  |  |  |
| haemorrhagic transformation of a brain infarct; n (%) |  |  |  |  |
| Major extracranial bleeding; n (%) |  |  |  |  |
| Major bleeding (intracranial and extracranial); n (%) |  |  |  |  |
| Clinically relevant non-major bleeding; n (%) |  |  |  |  |

**Table A5: Serious Adverse Events (SAE)**

| **Number of Patients reporting at least one SAE and by type** | **Early n=** | **Standard n=** | **Total N=** |
| --- | --- | --- | --- |
| SAE (unrelated); n (%) |  |  |  |
| SAE (related); n (%) |  |  |  |

**Table A6: Subgroup analyses**

|  | **Early n=** | **Standard n=** | **Adjusted Risk Difference (95% CI)** | **p-value** | **Interaction test p-value** |
| --- | --- | --- | --- | --- | --- |
| Stroke severity (NIHSS)  at randomisation |  |  |  |  |  |
| 0-4; n (%) |  |  |  |  |  |
| 5-10; n (%) |  |  |  |  |  |
| 11-15; n (%) |  |  |  |  |  |
| 16-21; n (%) |  |  |  |  |  |
| >21; n (%) |  |  |  |  |  |
| Age (years) |  |  |  |  |  |
| <75; n (%) |  |  |  |  |  |
| ≥75; n (%) |  |  |  |  |  |
| Sex |  |  |  |  |  |
| Female; n (%) |  |  |  |  |  |
| Male; n (%) |  |  |  |  |  |
| Use of anticoagulation at the time of qualifying acute ischaemic stroke |  |  |  |  |  |
| No; n (%) |  |  |  |  |  |
| Yes; n (%) |  |  |  |  |  |
| Atrial fibrillation previously known before qualifying acute ischaemic stroke |  |  |  |  |  |
| No; n (%) |  |  |  |  |  |
| Yes; n (%) |  |  |  |  |  |
| Diabetes type 1 or 2, known prior to stroke or diagnosis |  |  |  |  |  |
| No; n (%) |  |  |  |  |  |
| Yes; n (%) |  |  |  |  |  |
| Known chronic kidney disease |  |  |  |  |  |
| No; n (%) |  |  |  |  |  |
| Yes; n (%) |  |  |  |  |  |
| Reperfusion therapy |  |  |  |  |  |
| none; n (%) |  |  |  |  |  |
| intravenous thrombolysis alone; n (%) |  |  |  |  |  |
| intravenous thrombolysis and mechanical thrombectomy; n (%) |  |  |  |  |  |
| mechanical thrombectomy alone; n (%) |  |  |  |  |  |
