# Supplementary material for: Optimal timing of anticoagulation after acute ischaemic stroke with atrial fibrillation (OPTIMAS): statistical analysis plan for a randomised controlled trial
Source: Trials. 2025 Feb 19;26:58. doi: 10.1186/s13063-025-08761-6 (PMC11837694; doi:10.1186/s13063-025-08761-6)
Supplement: Supplementary file 2 — Additional file 2. Neuroimaging Substudy Analysis Plan. This file contains the analysis plan for the Neuroimaging substudy which will use the results of the data collected from the OPTIMAS trial for secondary analyses [file 13063_2025_8761_MOESM2_ESM.docx]

**Additional File 2 - Neuroimaging Substudy Analysis Plan**

**Optimal Timing of Anticoagulation After Stroke: A Randomised Controlled Trial: Neuroimaging-based Secondary Analysis Plan**

Jonathan Best^1^, Philip S. Nash^1^, James K. Ruffle^2^, David Doig^3^ and David J Werring^1^

1. Stroke Research Centre, Department of Brain Repair and Rehabilitation, UCL Queen Square Institute of Neurology, London, UK.
2. UCL Queen Square Institute of Neurology, University College London, London, UK.
3. Department of Neuroradiology, National Hospital for Neurology and Neurosurgery, University College London Hospitals NHS Trust, London, UK.

*Introduction and rationale*

When to start anticoagulation after acute ischaemic stroke in patients with AF is a frequent and important dilemma in stroke medicine. Several randomised trials have been undertaken with the aim of answering this question, including OPTIMAS, ELAN, TIMING and START (6-8, 40). However, determining the optimal timing of anticoagulation initiation for an individual patient will continue to require an individualised assessment of their risks of early recurrence and of ICH. The volume of infarcted tissue, on theoretical grounds and based on observational data (9), is an important determinant of the risk of haemorrhagic transformation. Clinical decision rules using a clinical assessment of stroke severity as a proxy for infarct size have been proposed (10,11), and stroke severity has been shown to influence anticoagulation timing in clinical practice (12). However, this approach is based only on observational data and expert opinion, and clinical stroke severity may underestimate infarct size (for example, in PCA occlusion) or overestimate it (for example, in brainstem infarction). There are also very few data on whether anticoagulation timing should be influenced by the presence of haemorrhagic transformation, or by imaging markers of cerebral small vessel disease, the major cause of spontaneous ICH and an important risk factor for symptomatic ICH following intravenous thrombolysis (13).

There is therefore a need for imaging-based analyses of data from randomised controlled trials. OPTIMAS is the largest ongoing trial in this area, with an original recruitment target of nearly 3,500 participants. Although OPTIMAS does not use imaging data to determine participant eligibility or anticoagulation timing, or obtain study-specific imaging, brain imaging performed for clinical reasons is collected for all participants, providing the opportunity for imaging-based secondary analyses. Here, we present the protocol for a neuroimaging-based sub-study in OPTIMAS, investigating the influence of infarct size and cerebral small vessel disease on the net benefit of early versus delayed anticoagulation with a DOAC after AF-associated acute ischaemic stroke.

The primary hypothesis to be tested by this sub-study is that infarct volume, assessed by MRI or CT, may modify the treatment effect of early anticoagulation with a DOAC after AF-associated ischaemic stroke, with respect to a primary outcome of all-cause stroke and systemic arterial embolism within 90 days of randomisation. We hypothesise that the treatment effect of early anticoagulation will be less favourable in patients with larger infarcts, due to higher rates of symptomatic haemorrhagic transformation. We will also test the hypothesis that a less favourable treatment effect will be seen in patients with haemorrhagic transformation of the initial infarct, and perform exploratory analyses including CT and MRI markers of cerebral small vessel disease.

methods and design

*Study setting*

The present study is a neuroimaging-based secondary analysis of OPTIMAS, a phase IV multicentre randomised controlled trial with an open-label intervention, blinded end-point adjudication, and hierarchical non-inferiority/superiority gatekeeper design, comparing a policy of early DOAC initiation, within 4 days of stroke onset, to delayed initiation, 7 to 14 days from onset, in patients with AF and acute ischaemic stroke. The primary outcome of OPTIMAS is a composite of recurrent ischaemic stroke, symptomatic ICH (including haemorrhagic transformation), unclassifiable stroke syndromes, and systemic arterial embolism incidence at 90 days post-randomisation. The target study sample size is 3,478 participants. OPTIMAS opened to recruitment in June 2019 and closed at the end of January 2024. It has recruited 3,648 participants from 100 stroke units in the United Kingdom.

*Patient population*

For each imaging exposure of interest, we will include all participants in OPTIMAS for whom the relevant imaging exposure can be assessed on imaging acquired prior to DOAC administration. We will exclude participants for whom the imaging exposure of interest cannot be measured due to absence of the relevant imaging modality or the failure to acquire images of diagnostic quality (as assessed by the OPTIMAS imaging rating team). For measurement of infarct size when participants have both MR and CT available to assess, we will segment the infarct in MRI owing its higher diagnostic accuracy, and when more than one CT is available we will choose the latest one prior to commencement of DOAC.

When stroke onset is unknown and cannot be estimated clinically with reasonable confidence, it should be determined in accordance with the main study protocol i.e. taken as the time the patient was last known to be well.

*Imaging data collection*

Any brain imaging performed for clinical reasons while a participant is enrolled in OPTIMAS is collected by the central study team, as well as imaging related to the qualifying stroke performed prior to randomisation. Sites send imaging via a secure online file transfer portal hosted at upload.optimas.org.uk, or exceptionally via posted CD if unable to use the online portal due to local technical or information governance requirements. Sites are required to pseudonymise imaging with the participant’s study ID prior to transfer. Files are sent in DICOM format. Once received, imaging is checked, backed up to the study imaging repository hosted by the UCL Research Data Storage service, and added to the study DICOM database (accessed via RadiAnt). Before infarct segmentation we will convert all the data to nifti format using the niftiReg software.

*Imaging data quality assurance*

The completeness of imaging data is monitored by the central study team. Sites report imaging performed to the central study team at discharge from the recruiting hospital site, and at the 90-day follow-up visit. These reports are regularly cross-checked against the imaging received, and sites contacted with prompts as needed.

On receipt, scans are checked for completeness by trained research fellows, and sites requested to resend incomplete imaging if needed. Scan quality will be assessed prior to analysis, and scans which are not of diagnostic quality excluded. When multiple eligible scans of the same modality and adequate technical quality are available, the last scan prior to DOAC administration will be preferred.

*Imaging rating procedures*

Imaging variables of interest will be rated by trained research fellows, using validated rating scales and consensus definitions when available (e.g. STRIVE, MARS) (14,15). To minimise inter-rater variability, before analysing study images, all raters will be required to rate a training set of thirty images of each modality for the primary exposures of interest and obtain an inter-rater reliability of >0.8 with the reference labels. The training set will be labelled by consultant neuroradiologists. To allow assessment of inter-rater reliability in the main study dataset, a random 10% of study images will be rated by at least two raters. Inter-rater reliability will be assessed using Krippendorff’s alpha for nominal or ordinal data, and the intraclass correlation coefficient for continuous variables. When manually segmenting CT infarcts, raters will use standard stroke windows (window 40, width 40) to identify the lesion, and these can be adjusted manually to optimally delineate the margins. If available we will segment the infarct on the thick axial slices, but we will use the thin slices if necessary for difficult cases. We will use ITKSnap version 3.6.0 to segment the infarcts.

*Automated segmentation of MR infarcts*

For the quantitative assessment of infarcts on MRI, we will utilise a deep learning segmentation model developed, validated out of sample, and described in detail elsewhere (16). In brief, this model uses the Swin UNet TRansformer (SwinUNETR) architecture with a custom loss function that optimises quantifying the volume and number of infarcts. This provides fidelity in delineating not only single infarcts but multifocal embolic ischaemia. It was trained on neuroimaging data from 10,463 patients, which includes 3563 infarct-positive diffusion-weighted imaging (B1000) cases acquired as part of routine clinical care at a tertiary neuroscience centre, and 6900 healthy controls. The model yields state-of-the-art performance evaluated on a large clinical held-out test set, with equitable performance (17) across patient demographics and all vascular territories. All model-derived lesion masks shall be reviewed and – if required – corrected by a trained reader under the supervision of an experienced neuroradiologist specialising in stroke imaging with more than 15 years of experience.

**Imaging variables of interest**

Our primary exposures of interest are:

- Infarct volume, measured manually through computer-assisted planimetry. Infarct volume will be measured on diffusion-weighted MRI sequences if available. If not, the last CT scan acquired prior to DOAC administration will be used.
- Presence of haemorrhagic transformation of the qualifying infarct classified by Heidelberg criteria as petechial (1a/HI1 or 1b/HI2), parenchymal (1c/PH1 or 2/PH2), or remote (3) (22).

Our secondary exposures of interest are:

- A semiquantitative classification of infarct size used previously in observational studies and in the ELAN trial (8,9,16), estimated using diffusion-weighted MRI if available, or the last eligible CT scan if not. Infarct size is classified as below:
- Minor: lesion <1.5cm maximum diameter in anterior or posterior circulation, or multiple tiny spots (“embolic shower”)
- Moderate: lesion in cortical superficial branch of anterior, middle or posterior cerebral arteries; in deep branch of middle cerebral artery; in internal borderzone territories; or two minor lesions
- Major: lesion involving complete territory of anterior, middle or posterior cerebral artery; lesion ≥ 1.5cm maximum diameter in brainstem or cerebellum; or two moderate lesions
- CT markers of cerebral small vessel disease:
- Leukoaraiosis measured through total simplified Fazekas scale (17)
- Presence of one or more lacunes according to STRIVE definition (14)
- Overall CT Small Vessel Disease score, comprising leukoaraiosis, lacunes, and atrophy (18)
- MRI markers of cerebral small vessel disease:
- Number of cerebral microbleeds on MRI, categorised as 0, 1, 2-4, and 5 or more.
- Presence of multiple cerebral microbleeds in strictly lobar distribution (suggestive of cerebral amyloid angiopathy)
- Presence of cortical superficial siderosis (focal or disseminated)
- White matter hyperintensity burden measured through total Fazekas score (19)
- Presence of one or more lacunes according to STRIVE definition
- Burden of MR-visible perivascular spaces in basal ganglia region, rated using an established five-level ordinal scale (20)
- Overall MRI Total Small Vessel Disease score, comprising leukoaraiosis, white matter hyperintensities, basal ganglia perivascular spaces and cerebral microbleeds (21)

**Sample size estimates**

The target sample size for OPTIMAS is 3,478 participants. In an interim analysis of the first 2,965 participants, all participants had a CT, 33% had a CT after 12 hours, 24% had an MRI before DOAC commencement, and 57% had either a CT acquired between 12 hours and DOAC commencement or an MRI. Interim data suggests a primary outcome rate of 4.3% (7). Allowing a conservative 5% margin for missing or poor-quality imaging, we estimate a sample size of 1,883 participants (81 events) for analyses of infarct volume, infarct size and haemorrhagic transformation, 790 participants (34 events) for analyses of MRI markers of cerebral small vessel disease, and 3,304 participants (142 events) for analyses of CT markers of cerebral small vessel disease. Owing to the fixed sample size, formal power calculations were not appropriate.

**Statistical analysis**

For each imaging variable of interest, we will test for modification of the effect of treatment allocation (early or delayed DOAC initiation) by including the imaging variable as an interaction term with the independent variable representing treatment allocation (based on the intention-to-treat principle) in a mixed-effects logistic regression model with the occurrence of the primary study outcome as the dependent variable. If there is a significant difference in NIHSS between the main trial groups, we will consider adjusting for stroke severity (NIHSS) at randomisation. We will adjust for clustering by including sites as random intercept terms. An interaction with p-value <0.05 will be accepted as statistically significant. We will not adjust for multiple comparisons, but significant interactions for our secondary exposures of interest will be treated as hypothesis-generating (23). We will not impute missing exposures of interest but will include in each analysis all participants for whom the relevant exposure of interest is available.

Additional secondary analyses may include:

- A sensitivity analysis excluding participants with CT imaging acquired between before 24 hours of stroke onset
- Sensitivity analyses around infarct size and volume assessed using CT only or MRI only
- Analysis of exposures of interest with respect to individual components of the primary study outcome (e.g. ischaemic stroke, ICH)
- The feasibility and reliability of automated approaches to the measurement of infarct volume

**Discussion**

Based on methodological recommendations for secondary analyses of clinical trials, our imaging variables of interest are divided into primary and secondary exposures (23). Primary exposures are those for which a subgroup analysis is well-motivated by external evidence, and the results of which are intended to influence clinical practice; whereas analyses of secondary exposures are less strongly supported by external evidence and are intended to be hypothesis-generating.

We chose infarct volume measured by computer-assisted planimetry as our primary exposure of interest for CT and MRI sub-studies as the volume of infarcted tissue is thought on theoretical grounds to be a key determinant of the risk of haemorrhagic transformation, and infarct size has been associated with risk of intracranial haemorrhage and recurrent ischaemic stroke in a number of observational studies of patients initiating anticoagulation after AF-associated stroke (4). Although time-consuming, planimetry is the current gold standard for the measurement of infarct volume (24), with excellent inter-rater reliability between trained raters (25). Assessing infarct size as volume, a continuous variable, gives the greatest statistical power to detect an interaction and determine whether infarct size should influence anticoagulation timing (26). Recognising the difficulty in manually measuring infarct volume in clinical practice, we include a semiquantitative classification of infarct size as a secondary outcome for both sub-studies, and a pooled analysis with the ELAN trial, which uses this classification, is planned. In the future, improved image segmentation techniques may allow the automated measurement of infarct volume in clinical practice.

Haemorrhagic transformation is relatively common after cardioembolic stroke and often delays anticoagulation (30,31). Participants with severe parenchymal or remote haemorrhagic transformation (grade 2 or 3 by Heidelberg classification) are excluded from OPTIMAS on safety grounds. However, minor haemorrhagic transformation (Heidelberg grade 1) is not clearly associated with worse clinical outcome (32), and has not yet been shown to affect the risk-benefit balance of early anticoagulation. As haemorrhagic transformation is anticipated to be a rare baseline characteristic in the study sample, we will investigate this question by combining CT and MRI data - the Heidelberg classification has been proposed to apply to both (22). We will perform sensitivity analyses to address the possibility that the prevalence and significance of haemorrhagic transformation may vary according to imaging modality, given the very high sensitivity of blood-sensitive MRI sequences.

To our knowledge, whether neuroimaging markers of cerebral small vessel disease should influence the timing of anticoagulation initiation after ischaemic stroke is largely unstudied. It is clear that cerebral small vessel disease is the main cause of intracerebral haemorrhage overall, and likely also accounts for the majority of intracerebral haemorrhage in patients taking oral anticoagulants (27). Although haemorrhagic transformation generally occurs within infarcted brain tissue, it might be hypothesised that the risk of haemorrhagic transformation would be higher in the presence of pre-existing vascular fragility. Of the various CT and MRI markers of cerebral small vessel disease, we selected the burden of cerebral microbleeds on MRI as a primary exposure of interest as it is the most prognostically significant marker of long-term ICH risk in patients taking antithrombotics (28), and also influences the risk of ICH after intravenous thrombolysis (29).

To reflect how imaging might be used in clinical practice to decide between early and more delayed anticoagulation, we include only participants with imaging acquired before commencement of anticoagulation. We exclude imaging acquired after DOAC initiation, as this could affect some imaging features, notably the presence of haemorrhagic transformation and cerebral microbleeds. We will also consider excluding CT scans acquired fewer than 6 hours from stroke onset, as the growth of the ischaemic core and the associated development of cytotoxic oedema and CT hypodensity occurs over hours (33). We do not have an early limit on the timing of MRI, as changes on diffusion-weighted MRI appear within minutes of stroke symptom onset (34,35), although a relatively limited increase in lesion size may occur over the first 72 hours (36).

**References**

**­**1. Sposato LA, Cipriano LE, Saposnik G, Ruíz Vargas E, Riccio PM, Hachinski V. Diagnosis of atrial fibrillation after stroke and transient ischaemic attack: a systematic review and meta-analysis. Lancet Neurol. 2015 Apr;14(4):377–87.

2. Hart RG, Pearce LA, Aguilar MI. Meta-analysis: antithrombotic therapy to prevent stroke in patients who have nonvalvular atrial fibrillation. Ann Intern Med. 2007 Jun 19;146(12):857–67.

3. Ruff CT, Giugliano RP, Braunwald E, Hoffman EB, Deenadayalu N, Ezekowitz MD, et al. Comparison of the efficacy and safety of new oral anticoagulants with warfarin in patients with atrial fibrillation: a meta-analysis of randomised trials. Lancet Lond Engl. 2014 Mar 15;383(9921):955–62.

4. Best JG, Cardus B, Klijn CJM, Lip G, Seiffge DJ, Smith EE, et al. Antithrombotic dilemmas in stroke medicine: new data, unsolved challenges. J Neurol Neurosurg Psychiatry. 2022 Sep 1;93(9):939–51.

5. Seiffge DJ, Werring DJ, Paciaroni M, Dawson J, Warach S, Milling TJ, et al. Timing of anticoagulation after recent ischaemic stroke in patients with atrial fibrillation. Lancet Neurol. 2019 Jan 1;18(1):117–26.

6. Oldgren J, Åsberg S, Hijazi Z, Wester P, Bertilsson M, Norrving B, et al. Early Versus Delayed Non–Vitamin K Antagonist Oral Anticoagulant Therapy After Acute Ischemic Stroke in Atrial Fibrillation (TIMING): A Registry-Based Randomized Controlled Noninferiority Study. Circulation. 2022 Oct 4;146(14):1056–66.

7. Best JG, Arram L, Ahmed N, Balogun M, Bennett K, Bordea E, et al. Optimal timing of anticoagulation after acute ischemic stroke with atrial fibrillation (OPTIMAS): Protocol for a randomized controlled trial. Int J Stroke Off J Int Stroke Soc. 2022 Jun;17(5):583–9.

8. Fischer U, Trelle S, Branca M, Salanti G, Paciaroni M, Ferrari C, et al. Early versus Late initiation of direct oral Anticoagulants in post-ischaemic stroke patients with atrial fibrillatioN (ELAN): Protocol for an international, multicentre, randomised-controlled, two-arm, open, assessor-blinded trial. Eur Stroke J. 2022 Jun 15;23969873221106044.

9. Paciaroni M, Agnelli G, Falocci N, Caso V, Becattini C, Marcheselli S, et al. Early Recurrence and Cerebral Bleeding in Patients With Acute Ischemic Stroke and Atrial Fibrillation: Effect of Anticoagulation and Its Timing: The RAF Study. Stroke. 2015 Aug;46(8):2175–82.

10. Kirchhof P, Benussi S, Kotecha D, Ahlsson A, Atar D, Casadei B, et al. 2016 ESC Guidelines for the management of atrial fibrillation developed in collaboration with EACTS. Eur Eur Pacing Arrhythm Card Electrophysiol J Work Groups Card Pacing Arrhythm Card Cell Electrophysiol Eur Soc Cardiol. 2016 Nov;18(11):1609–78.

11. Kimura S, Toyoda K, Yoshimura S, Minematsu K, Yasaka M, Paciaroni M, et al. Practical “1-2-3-4-Day” Rule for Starting Direct Oral Anticoagulants After Ischemic Stroke With Atrial Fibrillation: Combined Hospital-Based Cohort Study. Stroke. 2022 May;53(5):1540–9.

12. Munn D, Abdul-Rahim AH, Fischer U, Werring DJ, Robinson TG, Dawson J. A survey of opinion: When to start oral anticoagulants in patients with acute ischaemic stroke and atrial fibrillation? Eur Stroke J. 2018 Dec;3(4):355–60.

13. Best JG, Jesuthasan A, Werring DJ. Cerebral small vessel disease and intracranial bleeding risk: Prognostic and practical significance. Int J Stroke. 2023 Jan 1;18(1):44–52.

14. Wardlaw JM, Smith EE, Biessels GJ, Cordonnier C, Fazekas F, Frayne R, et al. Neuroimaging standards for research into small vessel disease and its contribution to ageing and neurodegeneration. Lancet Neurol. 2013 Aug;12(8):822–38.

15. Gregoire SM, Chaudhary UJ, Brown MM, Yousry TA, Kallis C, Jäger HR, et al. The Microbleed Anatomical Rating Scale (MARS): reliability of a tool to map brain microbleeds. Neurology. 2009 Nov 24;73(21):1759–66.

16. Hatamizadeh A, Nath V, Tang Y, Yang D, Roth H, Xu D. Swin UNETR: Swin Transformers for Semantic Segmentation of Brain Tumors in MRI Images. 2022;arXiv:2201.01266. Available at: https://ui.adsabs.harvard.edu/abs/2022arXiv220101266H. Accessed January 01, 2022.

17. Carruthers R, Straw I, Ruffle JK, et al. Representational ethical model calibration. NPJ Digit Med 2022;5:170.

18. Paciaroni M, Agnelli G, Falocci N, Tsivgoulis G, Vadikolias K, Liantinioti C, et al. Early Recurrence and Major Bleeding in Patients With Acute Ischemic Stroke and Atrial Fibrillation Treated With Non-Vitamin-K Oral Anticoagulants (RAF-NOACs) Study. J Am Heart Assoc. 2017 Nov 29;6(12).

19. Swieten JC van, Hijdra A, Koudstaal PJ, Gijn J van. Grading white matter lesions on CT and MRI: a simple scale. J Neurol Neurosurg Psychiatry. 1990 Dec 1;53(12):1080–3.

20. Appleton JP, Woodhouse LJ, Adami A, Becker JL, Berge E, Cala LA, et al. Imaging markers of small vessel disease and brain frailty, and outcomes in acute stroke. Neurology. 2020 Feb 4;94(5):e439–52.

21. Fazekas F, Chawluk JB, Alavi A, Hurtig HI, Zimmerman RA. MR signal abnormalities at 1.5 T in Alzheimer’s dementia and normal aging. AJR Am J Roentgenol. 1987 Aug;149(2):351–6.

22. Potter GM, Chappell FM, Morris Z, Wardlaw JM. Cerebral perivascular spaces visible on magnetic resonance imaging: development of a qualitative rating scale and its observer reliability. Cerebrovasc Dis Basel Switz. 2015;39(3–4):224–31.

23. Staals J, Makin SDJ, Doubal FN, Dennis MS, Wardlaw JM. Stroke subtype, vascular risk factors, and total MRI brain small-vessel disease burden. Neurology. 2014 Sep 30;83(14):1228–34.

24. von Kummer R, Broderick JP, Campbell BCV, Demchuk A, Goyal M, Hill MD, et al. The Heidelberg Bleeding Classification. Stroke. 2015 Oct;46(10):2981–6.

25. Kent DM, Rothwell PM, Ioannidis JPA, Altman DG, Hayward RA. Assessing and reporting heterogeneity in treatment effects in clinical trials: a proposal. Trials. 2010 Aug 12;11:85.

26. van der Worp HB, Claus SP, Bär PR, Ramos LM, Algra A, van Gijn J, et al. Reproducibility of measurements of cerebral infarct volume on CT scans. Stroke. 2001 Feb;32(2):424–30.

27. Braun T, Pukropski J, Yeniguen M, El-Shazly J, Schoenburg M, Gerriets T, et al. Inter- and intra-rater reliability of computer-assisted planimetry in experimental stroke research. J Neurosci Methods. 2019 Jan 15;312:12–5.

28. Altman DG, Royston P. The cost of dichotomising continuous variables. BMJ. 2006 May 6;332(7549):1080.

29. Seiffge DJ, Wilson D, Ambler G, Banerjee G, Hostettler IC, Houlden H, et al. Small vessel disease burden and intracerebral haemorrhage in patients taking oral anticoagulants. J Neurol Neurosurg Psychiatry [Internet]. 2021 Mar 19 [cited 2021 Jul 12]; Available from: https://jnnp.bmj.com/content/early/2021/03/18/jnnp-2020-325299

30. Best JG, Ambler G, Wilson D, Lee KJ, Lim JS, Shiozawa M, et al. Development of imaging-based risk scores for prediction of intracranial haemorrhage and ischaemic stroke in patients taking antithrombotic therapy after ischaemic stroke or transient ischaemic attack: a pooled analysis of individual patient data from cohort studies. Lancet Neurol. 2021 Apr;20(4):294–303.

31. Charidimou A, Shoamanesh A, Wilson D, Gang Q, Fox Z, Jäger HR, et al. Cerebral microbleeds and postthrombolysis intracerebral hemorrhage risk Updated meta-analysis. Neurology. 2015 Sep 15;85(11):927–924.

32. Arboix A, Oliveres M, Massons J, Pujades R, García‐Eroles L. Early differentiation of cardioembolic from atherothrombotic cerebral infarction: a multivariate analysis. Eur J Neurol. 1999;6(6):677–83.

33. Paciaroni M, Bandini F, Agnelli G, Tsivgoulis G, Yaghi S, Furie KL, et al. Hemorrhagic Transformation in Patients With Acute Ischemic Stroke and Atrial Fibrillation: Time to Initiation of Oral Anticoagulant Therapy and Outcomes. J Am Heart Assoc. 2018 Nov 20;7(22):e010133.

34. Berger C, Fiorelli M, Steiner T, Schäbitz WR, Bozzao L, Bluhmki E, et al. Hemorrhagic transformation of ischemic brain tissue: asymptomatic or symptomatic? Stroke. 2001 Jun;32(6):1330–5.

35. Saver JL. Time Is Brain—Quantified. Stroke. 2006 Jan;37(1):263–6.

36. Yoneda Y, Tokui K, Hanihara T, Kitagaki H, Tabuchi M, Mori E. Diffusion-weighted magnetic resonance imaging: Detection of ischemic injury 39 minutes after onset in a stroke patient. Ann Neurol. 1999;45(6):794–7.

37. Hjort N, Christensen S, Sølling C, Ashkanian M, Wu O, Røhl L, et al. Ischemic injury detected by diffusion imaging 11 minutes after stroke. Ann Neurol. 2005;58(3):462–5.

38. Lansberg MG, O’Brien MW, Tong DC, Moseley ME, Albers GW. Evolution of Cerebral Infarct Volume Assessed by Diffusion-Weighted Magnetic Resonance Imaging. Arch Neurol. 2001 Apr 1;58(4):613–7.

39. Wardlaw JM. RADIOLOGY OF STROKE. J Neurol Neurosurg Psychiatry. 2001 Apr 1;70(90001):7i–11.

40. King BT, Lawrence PD, Milling TJ, Warach SJ. Optimal delay time to initiate anticoagulation after ischemic stroke in atrial fibrillation (START): Methodology of a pragmatic, response-adaptive, prospective randomized clinical trial. Int J Stroke Off J Int Stroke Soc. 2019 Dec;14(9):977–82.
