# Supplementary material for: Optimal timing of anticoagulation after acute ischaemic stroke with atrial fibrillation (OPTIMAS): statistical analysis plan for a randomised controlled trial
Source: Trials. 2025 Feb 19;26:58. doi: 10.1186/s13063-025-08761-6 (PMC11837694; doi:10.1186/s13063-025-08761-6)
Supplement: Supplementary file 3 — Additional file 3. OPTIMAS Trial Team and Oversight Committee Members. This file contains the full list of OPTIMAS trial team and oversight committee members including names, affiliations and role. Statistical Analysis Plan (SAP) Checklist v1.0 [file 13063_2025_8761_MOESM3_ESM.docx]

**Additional File 3 – OPTIMAS Trial Team and Oversight Committee Members**

| **Trial Management Group Membership** | |  |
| --- | --- | --- |
| Name | Affiliation | Role |
| Prof David Werring | University College London | Chief Investigator |
| Prof Hannah Cohen | University College London | Co-applicant, Consultant Haematologist and Professor of Haematology |
| Dr Martin James | University of Exeter | Co-applicant, Consultant Stroke Physician and Associate Professor |
| Prof Gregory Lip | University of Liverpool | Co-applicant, |
| Prof Nikola Sprigg | University of Nottingham | Co-applicant, |
| Nicholas Freemantle | University College London, CCTU | Statistical Oversight, Director of UCL CCTU |
| Rachel Hunter | University College London. | Senior Health Economist |
| Ekaterina Bordea | University College London, CCTU | Health Economist |
| Hakim-Moulay Dehbi | University College London, CCTU | Trial Statistician |
| Norin Ahmed | University College London, CCTU | Trial Statistician |
| Amalia Ndoutoumou | University College London, CCTU | Clinical Project Manager |
| Sue Massingham | University College London | Stroke Research Clinical Trial Coordinator |
| Tishok Vanniyasingam | University College London, CCTU | Data Manager |
| Aneet Gill | University College London, CCTU | Trial Manager |
| Archana Jayanthi | University College London, CCTU | Trial Manager |
| Hannah Sims | University College London, CCTU | Trial Manager |
|  |  |  |
| **Trial Steering Committee Membership** | |  |
| Name | Affiliation | Role |
| Prof Gary Ford | University of Oxford | Professor of Stroke Medicine, Chair |
| Prof Christopher Price | Newcastle University | Professor of Stroke and Applied Health Research |
| Rosamund Yu | University College London | Patient Representative |
| Prof Stefan Engelter | Stroke Centre, University Hospital Basel, Switzerland | Non-Independent Member |
| Prof Bo Norrving | University of Liverpool, UK | Non-Independent Member |
| Dr Martin James | University of Exeter | Non-Independent Member |
| Prof Gregory Lip | University of Liverpool | Non-Independent Member |
| Prof Hannah Cohen | University College London | Non-Independent Member |
| Prof David Werring | University College London | Observer |
| Nicholas Freemantle | University College London, CCTU | Observer |
| Rachel Hunter | University College London. | Observer |
| Ekaterina Bordea | University College London, CCTU | Observer |
| Hakim-Moulay Dehbi | University College London, CCTU | Observer |
| Norin Ahmed | University College London, CCTU | Observer |
| Philip Nash | University College London | Observer |
| Amalia Ndoutoumou | University College London, CCTU | Observer |
| Sue Massingham | University College London | Observer |
| Tishok Vanniyasingam | University College London, CCTU | Observer |
| Aneet Gill | University College London, CCTU | Observer |
| Archana Jayanthi | University College London, CCTU | Observer |
| Hannah Sims | University College London, CCTU | Observer |
| Shannon Amolis | British Heart Foundation | Funder representative (observer) |
|  |  |  |
| **Independent Data Monitoring Committee Membership** | |  |
| Name | Affiliation | Role |
| Dr John Bamford | University of Leeds | Consultant Neurologist, Chair |
| Prof John Camm | St George's Medical School | Professor of Clinical Cardiology |
| Prof Tim Clayton | The London School of Hygiene and Tropical Medicine | Professor in Applied Medical Statistics |
| Dr Ander Cohen | King’s College London; Guy’s and St Thomas’ Hospitals NHS Foundation Trust | Consultant Vascular Physician |
| Prof Karin Klijn | Radboud University, Nijmegen, The Netherlands | Professor of Neurology |
| Prof David Werring | University College London | Chief Investigator |
| Nicholas Freemantle | University College London, CCTU | Professor of Clinical Epidemiology & Biostatistics, Director of CCTU |
| Hakim-Moulay Dehbi | University College London, CCTU | Unblinded Trial Statistician |
| Norin Ahmed | University College London, CCTU | Trial Statistician |
| Philip Nash | University College London | Clinical Fellow |
| Amalia Ndoutoumou | University College London, CCTU | Clinical Project Manager |
| Sue Massingham | University College London | Clinical Trial Coordinator |
| Tishok Vanniyasingam | University College London, CCTU | Data Manager |
| Aneet Gill | University College London, CCTU | Trial Manager |
| Archana Jayanthi | University College London, CCTU | Trial Manager |
| Hannah Sims | University College London, CCTU | Trial Manager |
|  |  |  |
| **Independent Event Adjudication Committee Membership** | |  |
| Name | Affiliation | Role |
| Dr Elizabeth Warburton | Addenbrookes’ Hospital and University of Cambridge | Consultant Stroke Physician, Chair |
| Prof Craig Smith | University of Manchester | Professor of Stroke Medicine |
| Dr Alastair Webb | John Radcliffe Hospital and University of Oxford | Consultant Neurologist |
| Philip Nash | University College London | Clinical Fellow |
| Sue Massingham | University College London | Clinical Trial Coordinator |
|  |  |  |
| **OPTIMAS Principle Investigators** | |  |
| Name | Affiliation | Role |
| Dr Fiona Humphries | UCLH - University College London Hospitals NHS Foundation Trust | Principle Investigators |
| Dr Thomas Harrison | Royal London - Barts Health NHS Trust | Principle Investigators |
| Dr Lakshmanan Sekaran | Luton and Dunstable - Luton and Dunstable Hospital NHS Foundation Trust | Principle Investigators |
| Dr Mohit Bhandari | Watford - West Hertfordshire Hospitals NHS Trust | Principle Investigators |
| Dr Madana Meegada | Broomfield - Mid Essex Hospital Services NHS Trust | Principle Investigators |
| Dr Manohar Kini | Southend - Southend University Hospital NHS Foundation Trust | Principle Investigators |
| Dr Ashok Mathews | Romford - Barking, Havering and Redbridge University Hospitals NHS Trust | Principle Investigators |
| Dr Rajaram Bathula | Northwick Park - London North West University Healthcare NHS Trust | Principle Investigators |
| Dr Soma Banerjee | Charing Cross - Imperial College Healthcare NHS Trust | Principle Investigators |
| Dr James Teo | Kings Colledge Hospital - King's College Hospital NHS Foundation Trust | Principle Investigators |
| Dr Udayaraj Umasankar | Lewisham Hospital - Lewisham and Greenwich NHS Trust | Principle Investigators |
| Dr Liqun Zhang | St Georges Hospital - St Georges Hospital NHS Trust | Principle Investigators |
| Dr Amit Kishore | Salford Royal - Salford Royal NHS Foundation Trust | Principle Investigators |
| Dr Aravind Manoj | Royal Liverpool - Royal Liverpool and Broadgreen University Hospitals NHS Trust | Principle Investigators |
| Dr David Hargroves | William Harvey - East Kent Hospitals University NHS Foundation Trust | Principle Investigators |
| Dr Saidu Abubaker | QE, The Queen Mother - East Kent Hospitals University NHS Foundation Trust | Principle Investigators |
| Dr Don Sims | Queen Elizabeth University Hospital - University Hospitals Birmingham NHS Foundation Trust | Principle Investigators |
| Dr Walee Sayed | Wrexham Maelor - Betsi Cadwaladr University Health Board | Principle Investigators |
| Dr Richard Marigold | Southampton General - University Hospital Southampton NHS Foundation Trust | Principle Investigators |
| Dr Hemsley Emsley | Royal Liverpool - Royal Liverpool and Broadgreen University Hospitals NHS Trust | Principle Investigators |
| Dr Naweed Sattar | Sunderland Royal - South Tyneside and Sunderland NHS Foundation Trust | Principle Investigators |
| Dr Aaizza Naqvi | Royal Hallamshire - Sheffield Teaching Hospitals NHS Foundation Trust | Principle Investigators |
| Dr Luke Bridge | York District General - York Teaching Hospital NHS Foundation Trust | Principle Investigators |
| Dr Stuart Maguire | Leeds General Hospital - Leeds Teaching Hospitals NHS Trust | Principle Investigators |
| Dr Samer Al-Hussayni | James Cook University Hospital - South Tees NHS Foundation Trust | Principle Investigators |
| Dr Brendan Affley | St Peters Hospital - Ashford and St Peters Hospitals NHS Foundation Trust | Principle Investigators |
| Dr David Eveson | Leicester Royal Infirmary - University Hospitals of Leicester NHS Trust | Principle Investigators |
| Dr Tim England | Royal Derby - Derby Teaching Hospitals NHS Foundation Trust | Principle Investigators |
| Dr Abul Azim | West Suffolk Hospital - West Suffolk Hospital NHS Foundation Trust | Principle Investigators |
| Dr Kelvin Kong | Lincoln County - United Lincolnshire Hospitals Trust | Principle Investigators |
| Dr Eoin O’Brien | Addenbrookes - Cambridge University Hospitals NHS Foundation Trust | Principle Investigators |
| Dr Jahanzeb Rehan | Kings Mill Hospital - Sherwood Forest Hospitals NHS Foundation Trust | Principle Investigators |
| Dr David Eveson | Nottingham University Hospitals - Nottingham University Hospitals NHS Trust | Principle Investigators |
| Dr Kiruba Nagaratnam | Royal Berkshire - Royal Berkshire NHS Foundation Trust | Principle Investigators |
| Dr Nigel Smyth | Royal Hampshire - Hampshire Hospitals NHS Foundation Trust | Principle Investigators |
| Dr Toby Black | Salisbury District Hospital - Salisbury NHS Foundation Trust | Principle Investigators |
| Dr John France | Torbay and Southern Devon Health and Care NHS Trust | Principle Investigators |
| Dr Maria Tuna | Wycombe Hospital - Buckinghamshire Healthcare NHS Trust | Principle Investigators |
| Dr Manju Krishnan | Morriston Hospital - Swansea Bay University Health Board | Principle Investigators |
| Dr Yousif Behnam | Milton Keynes - Milton Keynes University Hospital NHS Foundation Trust | Principle Investigators |
| Dr Kamy Thavanesan | Royal Bournemouth - University Hospitals Dorset NHS Foundation Trust | Principle Investigators |
| Dr Martin James | Royal Devon and Exeter - Royal Devon and Exeter NHS Foundation Trust | Principle Investigators |
| Dr Katja Adie | Royal Cornwall - Royal Cornwall Hospitals NHS Trust | Principle Investigators |
| Dr Dumin Karunatilake | Musgrove Park - Taunton and Somerset NHS Foundation Trust | Principle Investigators |
| Dr Alex Shah | Derriford - University Hospitals Plymouth NHS Trust | Principle Investigators |
| Dr Khalid Rashed | Yeovil District - Yeovil District Hospital NHS Foundation Trust | Principle Investigators |
| Dr Raj Shekhar | Queen Elizabeth Hospital - Queen Elizabeth Hospital, King's Lynn, NHS Foundation Trust | Principle Investigators |
| Dr Lukuman Gbadamoshi | Royal United - Royal United Hospitals Bath NHS Foundation Trust | Principle Investigators |
| Dr C Somasbekar | The Grange University Hospital - Aneurin Bevan University Health Board | Principle Investigators |
| Dr Syed Abid Raza | Bronglais General - Hywel Dda University Health Board | Principle Investigators |
| Dr Christopher James | Withybush General - Hywel Dda University Health Board | Principle Investigators |
| Dr Pagadala Sridhar | Glanwilli General - Hywel Dda University Health Board | Principle Investigators |
| Dr Senthil Kumar | Prince Philip Hospital - Hywel Dda University Health Board | Principle Investigators |
| Dr Brian Menezes | Arrowe Park - Wirral University Teaching Hospital NHS Foundation Trust | Principle Investigators |
| Dr Manohar Kini | Calderdale Hospital - Calderdale and Huddersfield NHS Foundation Trust | Principle Investigators |
| Dr Numan Khan | Bradford Royal Infirmary - Bradford Teaching Hospitals NHS Foundation Trust | Principle Investigators |
| Dr I Anwar | North Tees - North Tees and Hartlepool NHS Foundation Trust | Principle Investigators |
| Dr Mark Garside | Northumbria - Northumbria Healthcare NHS Foundation Trust | Principle Investigators |
| Dr Anthony Kenton | University Hospital of Coventry - University Hospitals Coventry & Warwickshire NHS Trust | Principle Investigators |
| Dr Suzanne Ragab | Poole Hospital - University Hospitals Dorset NHS Foundation Trust | Principle Investigators |
| Dr Harjit Bains | The John Radcliffe - Oxford University Hospitals NHS Foundation Trust | Principle Investigators |
| Dr Shahid Kausar | Russells Hall - Dudley Group NHS Foundation Trust | Principle Investigators |
| Dr Benjamin Jelley | University Hospital Wales - Cardiff & Vale | Principle Investigators |
| Dr Muhammad Hasan | Peterborough City - North West Anglia NHS Foundation Trust | Principle Investigators |
| Dr Michael McCormick | Craigavon - Southern Health and Social Care Trust | Principle Investigators |
| Dr Rajesh Saksena | Colchester - Colchester Hospital University NHS Foundation Trust | Principle Investigators |
| Dr Muhibbur Rahman Chowdhury | Ispwich - Ipswich Hospital NHS Trust | Principle Investigators |
| Prof Kausik Chatterjee | Countess of Chester - Countess of Chester Hospital NHS Foundation Trust | Principle Investigators |
| Dr Jim McIlmoyle | Royal Victoria Hospital - Belfast Health and Social Care Trust | Principle Investigators |
| Dr Harinath Chandrashekar | Leighton Hospital - Mid Cheshire Hospital NHS Foundation Trust | Principle Investigators |
| Dr Sevasti Ispoglou | Sandwell Hospital - Sandwell and West Birmingham Hospitals NHS Trust | Principle Investigators |
| Dr Breffni Keegan | South West Acute Hospital - Western Health and Social Care Trust | Principle Investigators |
| Dr Narayanamoorthi Saravanan | Fairfield Hospital - Pennine Care NHS Foundation Trust | Principle Investigators |
| Dr Gita Gramizadeh | Gloucester Royal - Gloucestershire Hospitals NHS Foundation Trust | Principle Investigators |
| Dr Nasar Ahmad | New Cross - Royal Wolverhampton Hospitals NHS Trust | Principle Investigators |
| Dr Bernard Esisi | Hull Royal Infirmary - Hull University Teaching Hospitals NHS Trust | Principle Investigators |
| Dr Venkatesan Srinivasan | Northampton General - Northampton General Hospital NHS Trust | Principle Investigators |
| Dr Mark Barber | University Hospital MonklandsNHS Lanarkshire | Principle Investigators |
| Prof M.J.Macleod | Aberdeen - NHS Grampian | Principle Investigators |
| Dr Mahmud Sajid | Chesterfield Royal - Chesterfield Royal Hospital NHS Foundation Trust | Principle Investigators |
| Dr Vera Cvoro | Victoria Hospital - NHS Fife | Principle Investigators |
| Dr Fergus Doubal | Royal Infirmary Edinburgh - NHS Lothian | Principle Investigators |
| Dr Anthony Byrne | Forth Valley - NHS Forth Valley | Principle Investigators |
| Dr Wai Meng Yu | Ninewells - NHS Tayside | Principle Investigators |
| Dr Bella Richard | Nevill Hall - Aneurin Bevan University Health Board | Principle Investigators |
| Dr Phillip Ferdinand | Royal Stoke - University Hospitals of North Midlands NHS Trust | Principle Investigators |
| Dr Jesse Dawson | Queen Elizabeth University Hospital - NHS NHS Greater Glasgow and Clyde | Principle Investigators |
| Dr Harald Proeschel | Dorset County - University Hospitals Dorset NHS Foundation Trust | Principle Investigators |
| Dr Michelle Davis | Royal Victoria Infirmary - Newcastle Upon Tyne NHS Foundation Trust | Principle Investigators |
| Dr Puneet Kakar | Epsom General - Epsom and St Helier University Hospitals NHS Trust | Principle Investigators |
| Dr Sunanda Mavinamne | Whiston - St Helens and Knowsley Teaching Hospital NHS trust | Principle Investigators |
| Dr Aleksandra Ekkert | Great Western - Great Western Hospitals NHS Foundation Trust | Principle Investigators |
| Dr Afzal Mahmood | Maidstone - Maidstone and Tunbridge Wells NHS Foundation Trust | Principle Investigators |
| Dr David Hargroves | Kent and Canterbury Hospital - East Kent Hospitals University NHS Foundation Trust | Principle Investigators |
| Dr Peter Anderton | Doncaster Royal Infirmary - Doncaster and Bassetlaw NHS Foundation Trust | Principle Investigators |
| Dr Thandar Soe | James Paget - James Paget University Hospitals NHS Foundation Trust | Principle Investigators |
| Dr Naval Shinh | Norfolk and Norwich - Norfolk and Norwich University Hospital NHS Foundation Trust | Principle Investigators |
| Dr Arun Singh | Royal Blackburn - East Lancashire Hospitals NHS Trust | Principle Investigators |
| Dr Imran Ashraf | Darent Valley - Dartford and Gravesham NHS Trust | Principle Investigators |
| Dr Sandeep Buddha | Southmead - North Bristol NHS Trust | Principle Investigators |
